# Measurement of the Parity-Violating Neutron Spin Rotation in ^4^*He*

**DOI:** 10.6028/jres.110.025

**Published:** 2005-06-01

**Authors:** C. D. Bass, J. M. Dawkins, D. Luo, A. Micherdzinska, M. Sarsour, W. M. Snow, H. P. Mumm, J. S. Nico, P. R. Huffman, D. M. Markoff, B. R. Heckel, H. E. Swanson

**Affiliations:** Indiana University/IUCF, Department, Department; National Institute of Standards and Technology, Gaithersburg, MD 20890; North Carolina State University/TUNL Department; University of Washington, Department, Department

**Keywords:** cold neutrons, liquid helium, nucleon-nucleon interaction, parity non-conservation, parity violation, spin rotation, superfluid helium, weak interaction, weak meson exchange amplitude

## Abstract

In the meson exchange model of weak nucleon-nucleon (NN) interactions, the exchange of virtual mesons between the nucleons is parameterized by a set of weak meson exchange amplitudes. The strengths of these amplitudes from theoretical calculations are not well known, and experimental measurements of parity-violating (PV) observables in different nuclear systems have not constrained their values. Transversely polarized cold neutrons traveling through liquid helium experience a PV spin rotation due to the weak interaction with an angle proportional to a linear combination of these weak meson exchange amplitudes. A measurement of the PV neutron spin rotation in helium (*φ*_PV_ (***n***,*α*)) would provide information about the relative strengths of the weak meson exchange amplitudes, and with the longitudinal analyzing power measurement in the ***p*** + *α* system, allow the first comparison between isospin mirror systems in weak NN interaction. An earlier experiment performed at NIST obtained a result consistent with zero: *φ*_PV_ (***n***,*α*) = (8.0 ±14(stat) ±2.2(syst)) ×10^−7^ rad / m[1]. We describe a modified apparatus using a superfluid helium target to increase statistics and reduce systematic effects in an effort to reach a sensitivity goal of 10^−7^ rad/m.

## 1. Introduction and Discussion

While the Standard Model has been remarkably successful in describing weak interactions between leptons, leptons and hadrons, and in the flavor-changing decays of hadrons, it has been difficult both experimentally and theoretically to test the Standard Model of the weak NN interaction. Since strong and electromagnetic amplitudes dominate at low energies, investigations are limited to parity-odd (PV) observables where weak currents must play a role. At low energies these processes are best described by an effective meson theory [2,3], where light virtual mesons such as the pion, rho, or omega are exchanged between nucleons. The PV NN interactions can then be characterized by six weak meson exchange amplitudes: *f*_π_, $h_{\rho }^{0},h_{\rho }^{1},h_{\rho }^{2},h_{\omega }^{0},\;$ and $h_{\omega }^{1}$, where superscripts refer to the isospin carried by the exchange meson.^1^ Of particular interest is the isovector pion exchange amplitude *f*_π_, since it is expected to arise primarily from weak neutral currents. Desplanques, Donoghue, and Holstein (DDH) employed a variety of theoretical techniques to determine a “reasonable range” and “best value” [3] for each of these amplitudes which now serve as valuable guides for experimental investigations. An effective field theory treatment of the weak NN interaction has also recently appeared [4].

One PV observable is the effect of the weak interaction on neutrons passing through matter, which is analogous to the optical rotation of polarized photons traveling through a “handed” medium [5]. A propagating neutron experiences a transverse rotation of its spin vector about its momentum axis—an effect which manifestly violates parity. The index of refraction of a medium in terms of the coherent forward scattering amplitude *f*(0) for a low-energy neutron [6] is

$$n=1+\left(2\pi /k_{\text{n}}^{2}\right)\;\rho \;f\;\left(0\right),$$ (1)

where ***k***_n_ is the incident neutron wave vector and *ρ* is the medium density. For zero nuclear spin media (e.g., ^4^He), the coherent forward scattering amplitude is the sum of two terms: a parity conserving (PC) term (*f*_PC_) that contains strong, electromagnetic, and weak interaction contributions, and a PV term (*f*_PV_) that contains only weak interaction contributions. The parity-odd *f*_PV_ is proportional to ***σ***_n_ · ***k***_n_(***σ***_n_ is the neutron spin vector) and so has opposite signs for (+) and (−) helicity states. Thus the indices of refraction of a medium for neutrons of opposite helicity states will differ.

As a neutron propagates through a medium, it accumulates different phases for its helicity states: *ϕ*_±_ = *ϕ*_PC_ ± *ϕ*_PV_, where *ϕ*_PC_ = *k z* (1 + 2π*ρ f*_PC_/*k*_n_^2^) and *ϕ*_PV_ = 2π*ρ z f*_PV_. Since a transversely polarized neutron is a linear combination of helicity states, it accumulates a phase difference as it propagates through a medium:

$$|{\hat{x}}_{+}\rangle =\frac{1}{\sqrt{2}}\left(|{\hat{z}}_{+}\rangle +|{\hat{z}}_{-}\rangle \right)\to \frac{1}{\sqrt{2}}\left(\left({\text{e}}^{i\phi_{+}}\right)|{\hat{z}}_{+}\rangle +\left({\text{e}}^{i\phi_{-}}\right)|{\hat{z}}_{-}\rangle \right).$$ (2)

While *ϕ*_PC_ contributes an overall phase factor dependent on *f*_PC_, *ϕ*_PV_ produces a rotation of the neutron spin vector about the momentum axis. This is the parity-violating neutron spin rotation:

$$\varphi_{\text{PV}}=\phi_{+}-\phi_{-}=2\phi_{\text{PV}}=4\pi \rho zf_{\text{PV}}.$$ (3)

This spin rotation can also be given in terms of the weak meson exchange amplitudes; for ^4^He, the calculated PV neutron spin rotation [7] is:

$$\varphi_{\text{PV}}\left(n,\alpha \right)=-\left(0.97f_{\pi }+0.32h_{\rho }^{0}-0.11h_{\rho }^{1}+0.22h_{\omega }^{0}-0.22h_{\omega }^{1}\right)\text{rad/m}.$$ (4)

The isospin mirror system of ***n*** + *α* is ***p*** + *α*, and its PV observable is the longitudinal analyzing power of polarized protons in helium (A_L_(***p***,*α*)). It has been measured measured and is given in terms of the weak meson exchange amplitudes [8]:

$$A_{\text{L}}\left(p,\alpha \right)=-\left(0.34f_{\pi }+0.14h_{\rho }^{0}-0.047h_{\rho }^{1}+0.059h_{\omega }^{0}-0.059h_{\omega }^{1}\right)\text{rad/m}.$$ (5)

Combining Eq. (4) and (5) yields an expression for the isovector pion exchange amplitude in terms of the other amplitudes and the PV observables:

$$f_{\pi }=-\left[0.51\varphi_{\text{PV}}\;(n,\alpha )+1.47A_{\text{L}}\;(p,\alpha )\right]+0.04h_{\rho }^{0}+0.13h_{\rho }^{1}-0.03h_{\omega }^{0}+0.21h_{\omega }^{1}.$$ (6)

Hence, a measurement of *φ*_PV_ (***n***,*α*) when combined with the measurement of (*A*_L_(***p***,*α*), can provide an important constraint on *f*_π_.

**Fig. 1:**
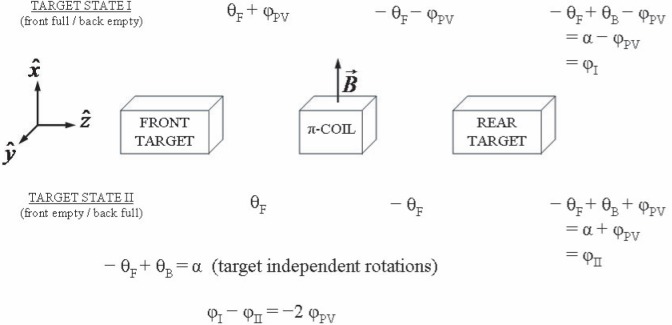
Progression of neutron spin as the beam propagates through the polarimeter in the $+\hat{z}$ direction. Rotations due to magnetic fields before and after the π-coil are indicated with subscripts “F” and “B” respectively. (Adapted from Ref. [1]).

In order to measure *φ*_PV_ (***n***,*α*), a neutron polarimeter^2^ is used which employs a crossed polarizer and alternating analyzer system. Between the polarizer/analyzer pair are two target chambers that are immediately upstream and downstream of a centrally-located solenoid, called a π-coil. One of the chambers is filled with liquid helium and the other is empty, although the contents of each can be remotely filled or emptied, so that two target states are possible. The field of the π-coil is aligned in the direction of the initial neutron polarization ( $\hat{x}\;$ direction) and is constructed so that when a neutron passes through it, its spin vector precesses 180° around the $\hat{x}\;$ axis. The analyzer is configured to modulate between the $+\hat{y}$ and $-\hat{y}$ directions. As a neutron beam travels through the polarimeter along the $\hat{z}$ direction (Fig. 1), the neutrons are initially polarized in $\hat{x}\;$ direction, pass through the front chamber, the π-coil, the back chamber, the analyzer, and into a segmented ^3^He ionization chamber detector [9] that measures the count rate associated with each analyzer state, *N*_+_ and *N*_−_ respectively. The neutron spin rotation is related to the count rate asymmetry [1] by:

$$\sin\;\varphi =\frac{N_{+}-N_{-}}{N_{+}+N_{-}}.$$ (7)

Unfortunately, the (PC) Larmor precession of a typical cold neutron (5 Å) due to the Earth’s magnetic field is approximately 10 rad/m, while *φ*_PV_(***n***,*α*) as calculated using the DDH “best values” is only (−0.1±1.15) × 10^−6^ rad/m. The experimental challenge is to distinguish tiny PV spin rotations from PC spin rotations. The π-coil precession reverses the $\hat{y}\;$ component of the neutron spin for rotations that occur in the front target. By modulating between target states, the $\hat{y}\;$ component of the PV spin rotation reverses sign, but target-independent PC rotations remain unaffected (to the extent that the helium does not alter the path length of the neutrons). Subtracting the measurements of the two target states eliminates the target-independent PC rotations and doubles the size of the PV signal.

All target-dependent PC rotations scale with the strength of residual fields, so they can be suppressed by reducing the background fields in the target region. These effects include the diamagnetic and optical potential differences between full and empty targets, as well as path length differences due to small-angle neutron scattering in liquid helium. The segmentation of the detector along the beam axis allows for a check on PC rotations, as the measurement of the rotation signal is a function of neutron energy: the PV rotation angle is independent of neutron energy [7], while PC rotations arising from residual fields scale as the inverse of neutron speed.

**Fig. 2:**
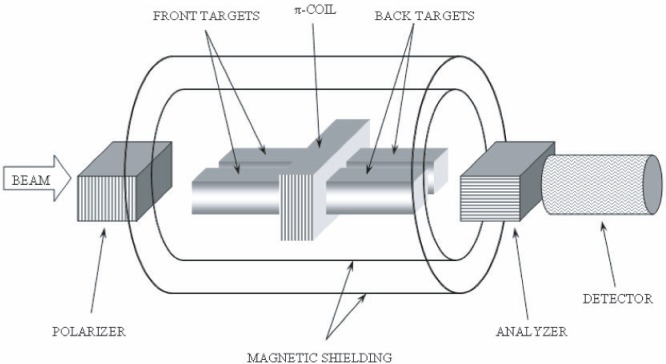
Schematic of experimental apparatus. After traveling through the polarizer, the beam is split into separate left and right halves, and then passes through front target chambers, the π-coil, back target chambers, the analyzer, and finally the detector. Magnetic shielding surrounds the cryostat (not shown) and target region.

In order to suppress noise due to reactor power fluctuations (on the order of 1 %), the neutron beam and targets are split into separate left and right sides (Fig. 2). The four chambers are filled in a pattern that ensures that the $\hat{y}\;$ component of the PV spin rotation in one sub-beam is opposite to that of the other sub-beam. The segmented ^3^He ionization chamber measures left and right side count rates independently, so by comparing the count rates from both sides, reactor power fluctuations can be eliminated from the signal.

The previous measurement [1] of *φ*_PV_(***n***,*α*) at the NIST Center for Neutron Research reached a sensitivity that was within a factor of 2 of the precision needed to provide new information about *f*_π_. The experiment was limited to about 12 days of data due to mechanical problems with the cryostat that surrounded the liquid helium targets, in addition to the relatively long fill and drain times (approximately 30 s to fill and 120 s to drain) of the target chambers needed to switch between polarimeter states, compared to the 600 s data acquisition time for any given polarimeter state.

Currently, the cryostat is being rebuilt and upgraded to allow the use of superfluid helium, which is denser than normal liquid helium and will increase the size of the PV neutron spin rotation by 20 %. Superfluid helium does not support the formation of bubbles within the target chambers, and more importantly, the small-angle neutron scattering cross-section of superfluid helium is smaller than normal liquid helium by a factor of 5 [11,12]. This will reduce systematic uncertainties associated with small-angle scattering to the level of 10^−8^ rad/m. Additionally, the targets have been redesigned so that the drain time is reduced (estimate 30 s to 60 s to drain), and this should increase available statistics by 10 % to15 %.

Since target-dependent systematic effects scale with the strength of the residual axial magnetic field, reduction of those fields are crucial. Previously, two coaxial shields built from Co-netic AA^3^ alloy along with field offset trim coils surrounding the cryostat reduced the residual magnetic fields in the target region to less than 1 nT. The addition of a third coaxial shield built from Cryoperm 10 alloy and positioned inside the cryostat could further decrease residual magnetic fields in the target to less than 0.5 nT. This will reduce the size of all target-dependent PC systematic uncertainties to the level of 10^−8^ rad/m or less. Finally, the NIST cold source has been upgraded since the last measurement, and beam fluence for the new experiment will be increased by a factor of approximately 1.5 [13].

We plan to finish upgrades on the polarimeter and move the apparatus back to NIST for a second run in 2005. Expecting to collect a much larger data set, we anticipate a precision of measuring *φ*_PV_(***n***,*α*) during the upcoming run on the order of 1 × 10^−7^ rad/m, which is an improvement of a factor of 5 over the previous measurement.

## Footnotes

1 There is a seventh weak meson exchange amplitude ${h^{\prime }}_{\rho }^{1}$, but it is estimated to be small compared to the others and is usually removed from further consideration.

2 This design was originally developed for measuring PV neutron spin rotation in solid targets by Ref. [9].

3 Certain commercial equipment, instruments or materials are identified in this paper to foster understanding. Such identification does not imply recommendation or endorsement by the National Institute of Standards and Technology, nor does it imply that the materials or equipment identified are necessarily the best available for the purpose.
